# Determining fuel moisture thresholds to assess wildfire hazard: A contribution to an operational early warning system

**DOI:** 10.1371/journal.pone.0204889

**Published:** 2018-10-04

**Authors:** Juan P. Argañaraz, Marcos A. Landi, Carlos Marcelo Scavuzzo, Laura M. Bellis

**Affiliations:** 1 Instituto de Diversidad y Ecología Animal (IDEA), CONICET-UNC and Facultad de Ciencias Exactas, Físicas y Naturales, Universidad Nacional de Córdoba, Córdoba, Argentina; 2 Instituto de Altos Estudios Espaciales "Mario Gulich", Comisión Nacional de Actividades Espaciales–Universidad Nacional de Córdoba, Falda del Cañete, Córdoba, Argentina; University of Vermont, UNITED STATES

## Abstract

Fuel moisture content (FMC) is an important fuel property for assessing wildfire hazard, since it influences fuel flammability and fire behavior. The relationship between FMC and fire activity differs among land covers and seems to be a property of each ecosystem. Our objectives were to analyze pre-fire FMC among different land covers and to propose a wildfire hazard classification for the Sierras Chicas in the Chaco Serrano subregion (Argentina), by analyzing pre-fire FMC distributions observed for grasslands, shrublands and forests and using percentiles to establish thresholds. For this purpose, we used a fire database derived from Landsat imagery (30 m) and derived FMC maps every 8 days from 2002 to 2016 using MODIS reflectance products and empirical equations of FMC. Our results indicated that higher FMC constrains the extent of wildfires, whereas at lower FMC there are other factors affecting their size. Extreme and high fire hazard thresholds for grasslands were established at FMC of 55% and 67% respectively, at 72% and 105% for forests and at 106% and 121% for shrublands. Our FMC thresholds were sensitive to detect extreme fire hazard conditions during years with high fire activity in comparison to average conditions. The differences in the distributions of pre-fire FMC among land covers and between ecosystems highlighted the need to locally determine land cover-specific FMC thresholds to assess wildfire hazard. Our wildfire hazard classification applied to FMC maps in an operational framework will contribute to improving early warning systems in the Sierras Chicas. However, moisture alone is not sufficient to represent true fire hazard in Chaco forests and the combination with other variables would provide better hazard assessments. These operational wildfire hazard maps will help to better allocation of fire protective resources to minimize negative impact on people, property and ecosystems. To the best of our knowledge, this is the first study analyzing pre-fire FMC over several fire seasons in a non-Mediterranean ecosystem, aiming at assessing wildfire hazard.

## Introduction

Wildfires are natural disturbances affecting the composition, structure and processes of landscapes worldwide [1]. However, current fire regimes in many areas are strongly influenced by humans that often increase the number of ignitions and fire frequency. Such departures from natural regimes threaten biodiversity and ecosystem dynamics as well as human life and infrastructure [2–4]. The moisture content of fuels (FMC) is an important fuel property for assessing wildfire hazard, since it influences fuel flammability and fire behavior [5–11]. As FMC increases, the flammability of fuels tends to decrease, because more energy is needed to evaporate water before burning organic tissues. In this context, operational estimations of FMC could be a valuable component of early warning systems [12], helping to minimize the negative effects of wildfires.

Given the relationship between FMC and fire activity, several studies have monitored FMC in fire prone ecosystems worldwide and their results indicate high variability among ecosystems and species. For instance, while fuelbed moisture content in grasslands (mix of live and dead standing fuels) ranges from < 30% to 300% in the Mediterranean Basin [13,14], and from 43% to 200% in South America [15], lower ranges between 28% to 88% were observed in grasslands of Southern Africa [16]. Additionally, different minimum live FMCs (LFMC) were observed in various shrubland ecosystems. While some Mediterranean shrubs show minimum values between 45% to 60% [13,14,17,18], other shrublands show minimums above 70% [15], 80% [19] and even above 90% or higher [20,21]. Likewise, LFMC varies among fire prone forests. In Australian schlerophyll forests LFMC ranges from 85% to 120% [19], while in semiarid forests of central Argentina, LFMC ranges from 60% to 160% [15] and in a dryer area of the same biome maximum values are lower than 60% [22].

When analyzing fire activity in relation to FMC, studies carried out in the Mediterranean ecosystems of North America observed that large fires occurred mostly with low values of LFMC (< 79%) (these values were obtained from field LFMC data collected every 2–3 weeks and then linearly interpolated to a daily resolution) [17,23]. Another study observed a considerable increase in the amount of burned area when monthly averaged LFMC lowered below 90% [24]. Instead, large fires in the Mediterranean Basin were observed in periods when FMC was lower than 35% in grasslands and lower than 84 to 110% in different shrub species (field samples were collected every 8 days, or every 16 days, but linearly interpolated at 8 day temporal resolution) [13]. Similarly, another study in Spain observed most fire activity in grasslands and shrublands when remotely sensed FMC was below 40% and 100%, respectively [25]. These results indicate the existence of FMC thresholds below which fire activity appears to be enhanced, but these thresholds seem to be system dependent.

Satellite imagery allow monitoring the temporal and spatial patterns of FMC at large scales. FMC can be estimated either by fitting empirical models relating field estimations of FMC with satellite derived data [15,17,19] or by inverting models of simulated reflectance (Radiative Transfer Models, RTM) [14,26,27]. MODIS data (Moderate-resolution Imaging Spectroradiometer) are widely used to derive FMC maps, since their spatial (500 m), spectral and temporal resolutions (8 days) are adequate to this purpose [14,15,19,28]. Ultimately, the challenge for stakeholders is to convert FMC maps into fire hazard maps in an operational way, in order to provide a practical tool for wildfire risk management systems at large scales.

Different strategies have been used to convert FMC into wildfire hazard based on empirical relationships between FMC and fire activity. Dennison et al. [17] found that large fires in the chaparral occurred with LFMC below 77%. Then, Peterson et al. [28] used this threshold and the thresholds of Weise et al. [29] to classify LFMC maps derived from MODIS as extreme (< 60%) and high fire hazard (60–77%). Jurdao et al. [25] analyzed the histograms of FMC before fires in Mediterranean grasslands and shrublands, and assigned the lowest ignition probability (IP) to the 90th percentile and the highest IP to the 10th percentile. The term wildfire hazard refers here to a combination of wildfire likelihood and intensity, as proposed by Scott et al. [30], although FMC is more a proxy of wildfire likelihood than a proxy of intensity. FMC is a good indicator of the ease of ignition (wildfire likelihood), but large fires occur with lower FMC [23,24] and those fires tend to have higher intensities and are more difficult to suppress [31].

According to the analyzed literature, the ranges of fuel moisture vary considerably among ecosystems and so does the relationship between FMC and fire activity. Even though a greater fire occurrence at lower values of FMC is out of discussion, evidence suggests that this relationship is a property of each ecosystem, and determining fuel moisture thresholds to assess wildfire hazard is a task that should be addressed for each system. The different FMC thresholds observed in relation to fire activity are probably related to a combination of variables acting at different scales. Structural and chemical properties also affect flammability at the plant level [20,32] and dead fuel loads and moisture affect flammability at the stand level [7,33]. These variables, along with weather conditions [23,34] and topography, interact affecting the relationship between FMC and fire occurrence in different ecosystems, becoming an emergent property at the landscape scale, as suggested by Yebra et al. [35]. So far, most prior research comparing FMC and fire occurrence has focused on Mediterranean ecosystems and little is known about other fire-prone ecosystems [35].

In central Argentina, the Sierras Chicas in the Chaco Serrano subregion is one of the areas most affected by fires, with more than 297,000 ha burned between 1999 and 2016 (i.e., 36.6% of total area) [36,37]. The high fire frequency and great number of large fires observed in the area potentially threaten more than 850,000 inhabitants. Moreover, almost one-half of the total number of buildings are located in the Wildland-Urban Interface (WUI), where their exposure to wildfires is higher due to the proximity of fuels [36,37]. In addition, fires are associated with the loss and degradation of relict forests, which derive in biodiversity loss [38–41] and deterioration of other ecosystem services [42,43].

At present, the existing early warning systems in the Sierras Chicas are based on: i) sparse point-based estimations of the fire danger indices from the Canadian Forest Fire Weather Index System [44], which might not have wide spatial representativeness due to the rough terrain, and ii) low spatial resolution daily maps (15 km) of McArthur's Fire Danger Index [45] implemented operationally using the Weather Research and Forecast (WRF) model [46]. The risk posed to the growing population and the fragile landscape urges the improvement of the current early warning systems solely based on the atmospheric conditions. Empirical FMC equations fitted relating field estimations of FMC and MODIS reflectance products (MYD09A1, 500-m pixel, 8-days) are available for the Sierras Chicas [15]. The operational implementation of these MODIS-derived FMC maps would be a great contribution to improve current warning systems. In order to properly use remotely sensed derived FMC to assess wildfire hazard in the Sierras Chicas, the relationship between FMC and fire activity must be established, since previous studies carried out in different fire-prone ecosystems worldwide observed different relationships between these variables.

In this study, we developed a land cover-specific wildfire hazard classification based on grassland fuelbed moisture content and shrubland and forest live fuel moisture content in the Sierras Chicas in the Chaco Serrano subregion. To accomplish this, we analyzed the pre-fire FMC distributions for different land covers: forests, shrublands and grasslands. In addition, we analyzed the relationship between pre-fire FMC and wildfire size in our study area. To the best of our knowledge, this is the first study analyzing pre-fire FMC over several fire seasons in a non-Mediterranean ecosystem, aiming at assessing wildfire hazard. Our research will contribute to developing operational fire hazard maps and improving existing early warning systems. Furthermore, our methods can be replicated in other ecosystems worldwide in order to identify specific FMC thresholds to assess fire hazard appropriately.

## Methods

Field surveys to collect reference data to derive the land cover map were conducted on public and private lands. F. Barri gave us permission to conduct field surveys on Vaquerías Natural Reserve, which belongs to the National University of Córdoba (Argentina). Field surveys carried out on private lands were conducted with permission of the owners. No permissions were necessary for Province Protected areas because we only visited public lands and we did not collect material. Our field studies did not involved endangered or protected species.

### Study area

**Fig 1:**
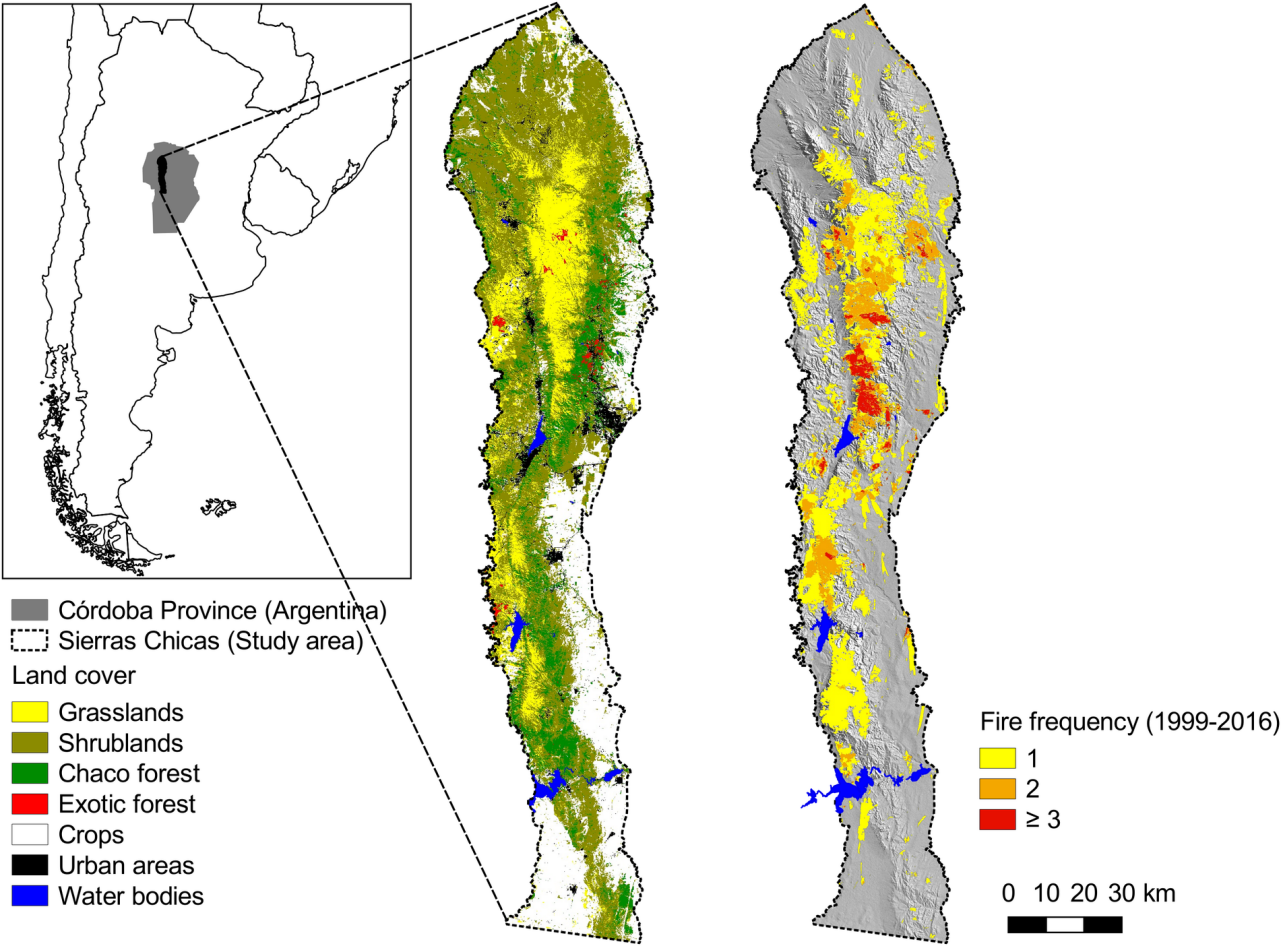
Fire frequency (1999–2016) and land cover in the Sierras Chicas in Córdoba province, Argentina.

**Fig 2:**
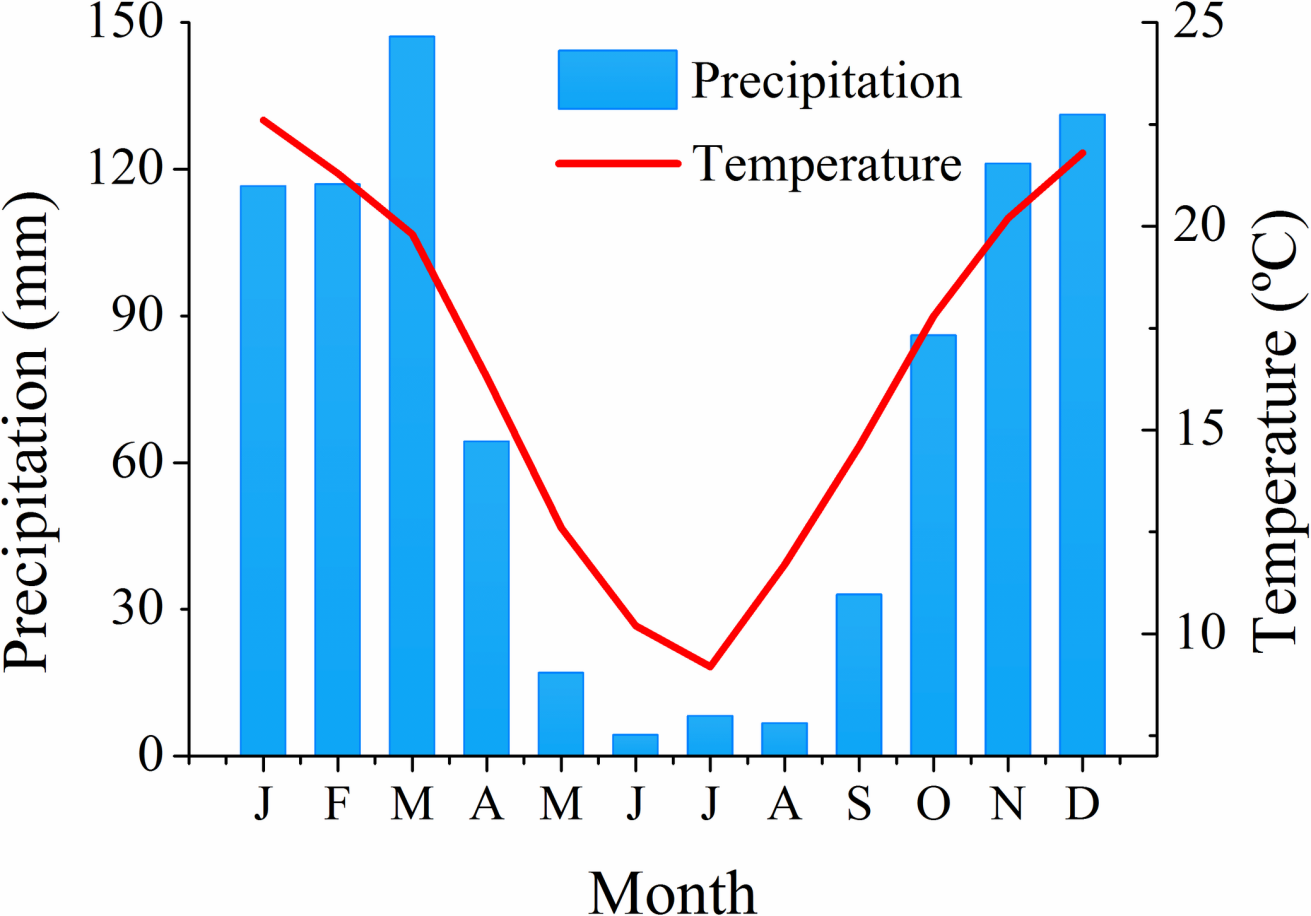
Mean monthly temperature and rainfall in the Sierras Chicas in Córdoba province, Argentina. Weather data (1999–2014) was provided by the National Weather Service of Argentina, Córdoba AERO weather station.

We conducted the study in the Sierras Chicas (810,000 ha) in Córdoba province, Argentina, which belongs to the Chaco Serrano subregion, in the southern portion of the seasonally dry forest of Gran Chaco (Fig 1). This mountain range stretches about 245 km from north to south (30° 20' S—32° 34' S) and 55km from east to west (64° 7' W—64° 45' W), with an altitudinal range between 500 and 1947 m a.s.l. The climate is temperate semiarid with a monsoonal rain regime, with a mean annual rainfall of 850 mm and a mean annual temperature of 17.3°C (National Meteorological Service of Argentina, data from the 1999–2014 period). Rainfall is concentrated between October and March (spring and summer; Fig 2). Winter is dry and mild, with relatively high temperatures in August and September, when most fires occur [36]. Between 1999 and 2016, nearly 297,000 ha burned (equivalent to 36.6% of our study area), with some areas burning up to three or more times in this period (Fig 1). The spatial pattern of fire frequency is driven by rainfall patterns, human presence, land cover and topography [36,37,47,48].

Vegetation consists of a mosaic of thorny semi-deciduous forests, shrublands, and grasslands (Fig 1). These vegetation physiognomies are represented along the elevation gradient, although in variable proportions. Chaco Serrano forests, dominated by *Lithraea molleoides*, are more frequent below 900 m. Their physiognomy is low, open to locally closed forest, with a total cover between 80 and 100%. Open areas have an open shrub layer and an herb layer [49]. Shrublands are dominated by *Vachellia caven* and *L*. *molleoides* appear as open and low to medium tall (2–4 m) shrublands with variable cover of an herbaceous layer [49] and are more frequent below 1300 m. Grasslands dominated by *Festuca hieronymi* are usually found above 900 m [50]. Recurrent fires turn forests into shrublands and grasslands, homogeneizing the landscape and increasing its flammability [51,52], which prevents the recovery of forests [53]. Most fires are caused by humans, either intentionally or accidentally. For instance, rural inhabitants use fire to promote forage re-growth during the dry season [40] and local governments set fire to reduce trash volume in open sky waste disposals. Also, some fires are related to tourism activities and arson.

More than 850,000 people live in the area, and almost half of the buildings (≈ 144,000) are located in WUI areas, where wildfire exposure is higher [37]. Moreover, estimations of population growth project an overall increase of 33% from 2010 to 2025 (National Institute of Statistics and Censuses of Argentina, INDEC), and according to the current patterns of urbanization, this will increase the WUI area and, consequently, the number of people and buildings at risk [37].

### Fuel moisture content modeling

FMC was estimated using MODIS surface reflectance products by applying empirical equations fitted for local vegetation [15]. Five 50 x 50-m plots were established in each of the three main vegetation types of the Sierras Chicas (15 plots in total): Chaco Serrano forests, shrublands and grasslands [50,54]. Each plot was sampled approximately every three weeks during the 2012 and 2013 fire seasons (June-December). Live fuel samples of forests and shrublands (terminal twigs and leaves) and whole fuelbed samples of grasslands (i.e. mix of live and dead fuels) (~200–400 g) were collected and transported to the laboratory in sealed bags where fresh weight (W_f_) was determined. Then, samples were oven-dried at 105°C until constant weight (≈ 72 hs) to determine dry weight (W_d_) [55] and FMC (%) was determined as: 100 * (W_f_−W_d_)/W_d_ [21]. In this paper we will use the term fuel moisture content (FMC) to refer to the fuelbed moisture content in the case of grasslands (our data and cited references) and to live fuel moisture content (LFMC) in the case of shrublands and forests.

The independent variables included fifteen spectral indices derived from MODIS land surface reflectance product MYD09A1 (500 m of spatial resolution; freely available at: http://daac.ornl.gov/MODIS/modis.shtml), which provides a composite image every 8 days. The indices were selected based on previous research relating field estimations of FMC and remote sensing data [19,35,56] and involved indices highlighting vegetation chlorophyll content (i.e., indices based on the visible and NIR bands) and vegetation water content (i.e., indices including the SWIR band) [57]. For each sampling year, a 31-image stack corresponding to the May-December period (days of year 121 to 361) was built and then spectral indices were calculated. Afterwards, the Savitsky-Golay filter was applied to smooth the typical noise of these time series [58,59], using a temporal window of ±3 MYD09A1 dates (24 days). Then, the field estimation of FMC was associated with the smoothed time series of the different spectral indices corresponding to the closest date of the MODIS product, before or after the sampling date. The relationship between the field-estimated FMC for each land cover type and the independent variables was analyzed using mixed linear models, obtaining the following empirical models for Chaco Serrano forests (R^2^ = 0.86), grasslands (R^2^ = 0.88), and shrublands (R^2^ = 0.57), [15]:

$$LFMC_{ChacoSerrano}=1.88Integral+246.39NDVI-63.06$$

$$FbMC_{Grasslands}=540.09EVI-31.16$$

$$LFMC_{Shrublands}=334.53EVI-305.98GVMI-7.05Integral+199.72$$

### Fuel moisture content estimation

The empirical models were used to estimate FMC maps for our study area every 8 days between 2002 and 2016 using MYD09A1 products. To select the appropriate model for each pixel, a land cover map is necessary. Two land cover maps were used for this analysis in order to account for land cover changes during the time span of our study. A land cover map derived from Landsat 5 TM images acquired in 1997 was available [60], and we derived a second land cover map for this study using data from 2013. We generated our land cover map by performing a multitemporal classification of Landsat 8 OLI images acquired on April 16 and August 6, 2013 (path/rows 229/81, 229/82). Imagery preprocessing included conversion of digital numbers to reflectance to the Top of the Atmosphere (TOA). We performed a supervised classification using Support Vector Machines and selecting the kernel Radial Basis Function. The C and γ parameters required for this kernel were determined using a 10-fold cross-validation performed via the package "e1071" [61] in R [62]. The ranges considered for these parameters were C ∈ [2^–5^, 2^15^] and γ ∈ [2^–15^, 2^3^], according to previous recommendations [63]. The reference data to train the classifier and to assess the accuracy of the land cover map were obtained from field surveys carried out in 2013 and from Google Earth images. We divided these data via stratified random sampling using land cover type as the strata, separating 70% to train the classifier and 30% to assess the accuracy of the map. The overall accuracy of this map was 93.8% (See error matrix and area of each land cover in S1 Table). To select the appropriate FMC model for each pixel, we needed the land cover map at the same spatial resolution than that of MODIS reflectance products. Both land cover maps (30-m spatial resolution) were resampled to match the resolution of MODIS reflectance products (500 m) using the majority filter, which is appropriate for categorical data. We used the land cover map of Zak [60] to calculate FMC for the fire seasons between 2002 and 2006 and the land cover map of 2013 for the fire seasons between 2007 and 2016.

In the same way as it was done to fit the empirical models, we created 31-image stacks for the predictor indices (NDVI, EVI, GVMI and Integral) using MYD09A1 products. Images corresponded to the period from May to December (days of year 121 to 361). The 31-image time series of each index were smoothed using the Savitsky-Golay filter [58,59], using a temporal window of ±3 MYD09A1 dates. Afterwards, we estimated FMC using the equations of Argañaraz et al. [15] according to the corresponding land cover type.

### Relationship between pre-fire FMC and fire size

To determine if fires of different sizes showed different pre-fire FMC values, we calculated the average FMC within the boundaries of burned polygons using zonal statistics (one fire = one sample). Pre-fire FMC values were extracted from the FMC maps corresponding to the date immediately before the dates when the fires began. For this analysis, we did not discriminate among land cover classes. We classified fire sizes in small (100–500 ha), medium (500–1,000 ha) and large fires (≥ 1,000 ha). Considering that one fire equals one sample in this analysis, we discarded fires in which there were no FMC data for more than 25% of their area (for instance, fires including more than 25% of cultivated lands, for which we do not have an equation to estimate FMC). We used Kruskal Wallis test to compare pre-fire FMC values among fires of different sizes, since preliminary tests indicated that variances were not homogeneous. Statistical analysis were performed with R [62]. We analyzed the relationship between pre-fire FMC and fire (S2 Table) size using quantile regression (deciles) with "quantreg" package [64].

### Wildfire hazard assessment based on pre-fire FMC

We calculated pre-fire FMC histograms for each land cover (S3 Table) in order to analyze if there were differences in the moisture content values before fires among grasslands, shrublands and Chaco Serrano forests. To accomplish this, we first intersected burned area polygons with land cover polygons. For instance, if a fire burned all the land cover types considered here, for that fire we got three intersections, one for grasslands, one for shrublands and one for Chaco Serrano forests. Then, for each intersection, we calculated the pre-fire FMC histogram, i.e., using the first FMC map available before the occurrence of each fire. Afterwards, we joined all the histograms of each land cover and analyzed the distribution of values. For this study, we only included fires larger than 100 ha, i.e., at least four MODIS pixels (25 ha each pixel). The fire database we used was derived from Landsat TM, ETM+ and OLI imagery (30-m spatial resolution) and the date of fire occurrence was determined using MODIS FIRMS, Córdoba Province data and newspapers [36].

Based on the distribution of pre-fire FMC values for each land cover, we established an FMC wildfire hazard classification discriminating four categories: Low, Moderate, High and Extreme fire hazard. To establish the FMC thresholds for each category we used percentiles, which have traditionally been used for similar purposes. For instance, the Canadian Forest Fire Weather Index System (CFWIS), one of the most widely used fire danger rating systems worldwide, needs to be locally calibrated to determine the class thresholds to asses fire danger, and the 90th and 97th percentile values of historical weather data are often used to determine the lower limits for the most hazardous classes [65,66].

Considering that a good wildfire hazard classification should include in its more hazardous categories most of the area burned, we used the following percentiles: P60 defined Extreme hazard, P85 defined High hazard, P97 defined Moderate hazard and FMC values higher than P97 defined Low fire hazard. The advantage of using percentiles is that different users can select other values to establish thresholds, whether they are more conservative or flexible, according to their preferences and needs. It is important to recall, though, that our fire hazard classification based on the distributions of pre-fire FMC does not intend to predict either the behavior of individual fires or fire suppression difficulties, but rather to assess potential fire activity for an area, as in the case of fire danger rating systems [65,67].

As a way of testing our wildfire hazard classification we compared our thresholds against three variables indicating expected moisture conditions: i) average of the mean FMC calculated for each land cover and date (every 8 days from May to December) from 2002 to 2015; ii) average of the mean FMC for years with high fire activity (burned area > 3% of total area): 2003, 2006, 2008, 2009, 2011 and 2013; and iii) average of the mean FMC for years with low fire activity (burned area < 1% of total area): 2004, 2007, 2010, 2012, 2014 and 2015. We also compared our classification against the moisture thresholds of Weise et al. [29] by calculating the percentage of burned pixels for each land cover that would have been classified in each hazard category. The desirable result would be that our classification includes a proportion of burned pixels in the hazardous categories (high and extreme) larger than the pre-existing classification.

## Results

We were able to analyze pre-fire FMC for 11 fire seasons (out of 15). Fire seasons of 2004, 2007 were not included because it was not possible to determine the dates of the fires; however, only five fires larger than 100 ha occurred, accounting for ~ 4,000 ha [36]. In 2015 and 2016, there were no fires larger than 100 ha in the Sierras Chicas. The burned area accounted by fires larger than 100 ha in the 11 fire seasons was 250,052 ha (equivalent to 31% of our study area, and 84% of total burned area between 1999 and 2016).

### Relationship between pre-fire FMC and fire size

**Fig 3:**
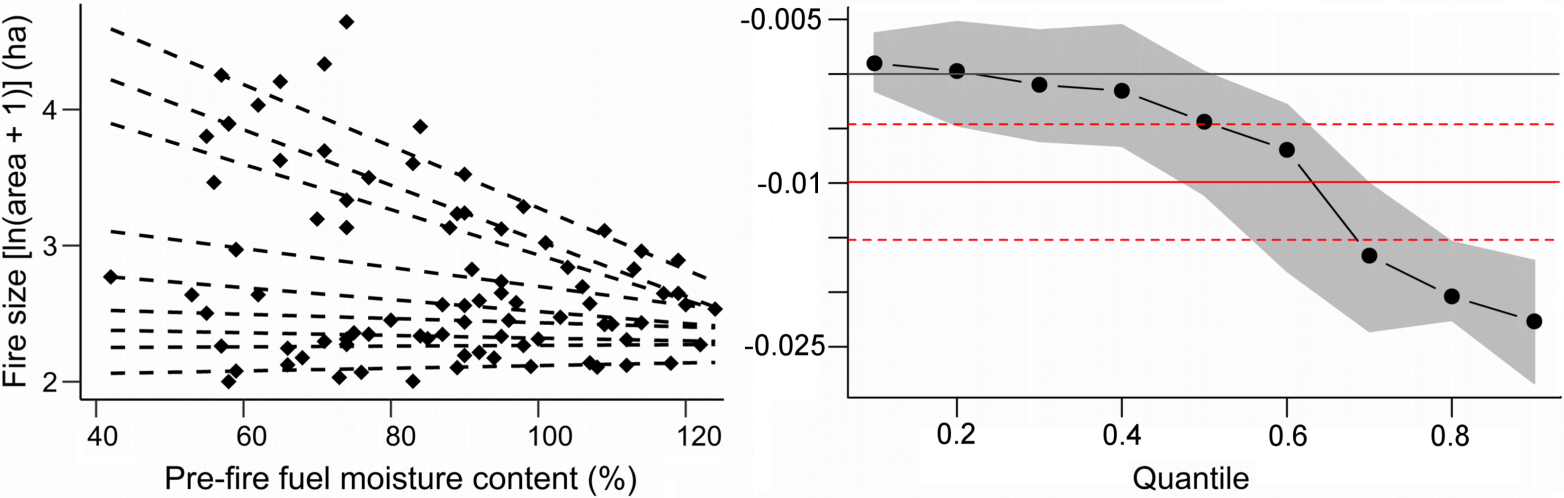
Scatter plot of fire size vs. pre-fire FMC (left) in the Sierras Chicas in Córdoba (Argentina). Data from 2002 to 2016 (n = 86). Dashed lines indicate quantile regressions fitted for quantiles between 0.1 and 0.9. Slope coefficients for quantiles (black dots) and their confidence intervals (grey area) are shown on the right plot. The red lines are the least squares estimate and its confidence interval.

The scatter plot of fire size vs. pre-fire FMC indicates that FMC is important for constraining the maximum sizes fires reach, but it has limited effect on the mean size (Table 1; Fig 3). Quantile regression estimates indicated a significant negative relationship between the upper 30% fire sizes and pre-fire FMC (quantiles ≥ 0.7: p < 0.05; Fig 3). Pseudo-R^2^ for these upper quantiles increases from 0.11 (0.7 quantile) to 0.25 (0.9 quantile). The comparison between pre-fire FMC and fire size showed significant differences between large fires (> 1000 ha) and small fires (100–500 ha) (Kruskal Wallis H = 8.44; P = 0.0037). Large fires occurred with median LFMC values around 74%; whereas small fires occurred with median values around 91%, which means a difference of 17 percentage points (Table 1). Intermediate-size fires (500–1000 ha) were not included in the analysis due to the low number of cases available (n = 9), after discarding those events with no information about the date of occurrence (3 cases) and those with LFMC estimations not reaching 75% of their area (3 cases).

**Table 1:** Summary statistics of average pre-fire FMC (one fire = one case) for different fire sizes in the Sierras Chicas in Córdoba, Argentina).

Fire size	N	Average	Minimum	Maximum	Median	Q1	Q3	Interquartile range
**Small**	52	90.3	52.6	124.2	91.1^a^	74.0	106.6	32.6
**Intermediate**	9	88.4	42.1	119.2	94.6	59.3	113.1	28.3
**Large**	25	76.6	55.2	108.8	73.8^b^	64.8	88.8	24.0

N: sample size. Q1: 1^st^ quartile, Q3: 3^rd^ quartile. Small fires: 100–500 ha; Intermediate: 500–1000 ha; Large: > 1000 ha. Different letters indicate statistical differences by Kruskal Wallis test, P < 0.05). Intermediate size fires were not included in this test due to the low number of cases available.

### Pre-fire FMC in different land covers

**Fig 4:**
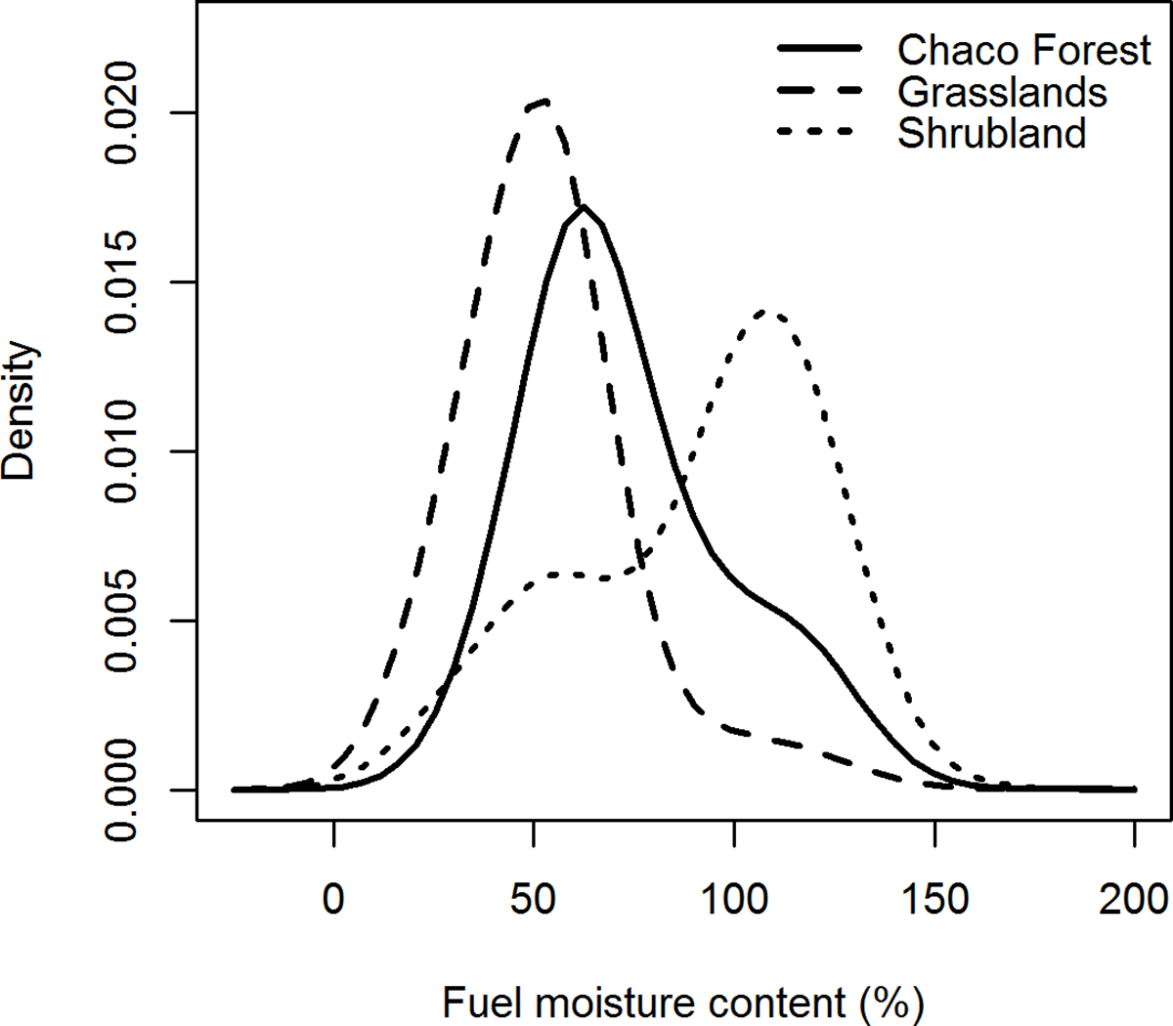
Pre-fire FMC density plots for dominant land covers in the Sierras Chicas in Córdoba, Argentina. Data from 2002–2016.

The distribution of pre-fire fuel moisture content values was different among land covers. Grasslands average fuelbed moisture content before fires was 53% and ninety percent of the burned area had FbMC values below 73% (Table 2; Fig 4). Chaco Serrano forests burned with average LFMC of 73% and ninety percent of the burned area had values below 114%. Shrublands burned with the highest LFMC values, with an average pre-fire LFMC of 91% (Table 2; Fig 4).

**Table 2:** Summary statistics of pre-fire FMC for different land covers in the Sierras Chicas in Córdoba, Argentina. Values represent fuelbed moisture content for grasslands and live fuel moisture content for forests and shrublands.

Land cover	Average (SD)	Minimum	Maximum	P50	P60	P85	P90	P97	N
**Grasslands**	53.2 (20.7)	6.2	182.7	52.3	54.8	66.7	73.2	110.6	4578
**Chaco forest**	73.1 (24.5)	8.1	164.9	66.5	72.0	104.5	114.1	125.1	1596
**Shrublands**	90.6 (30.6)	12.5	177.7	99.1	106.0	120.7	124.3	132.8	3304

SD: Standard deviation; N = number of pixels; P: percentiles

### Wildfire hazard classification based on FMC

**Fig 5:**
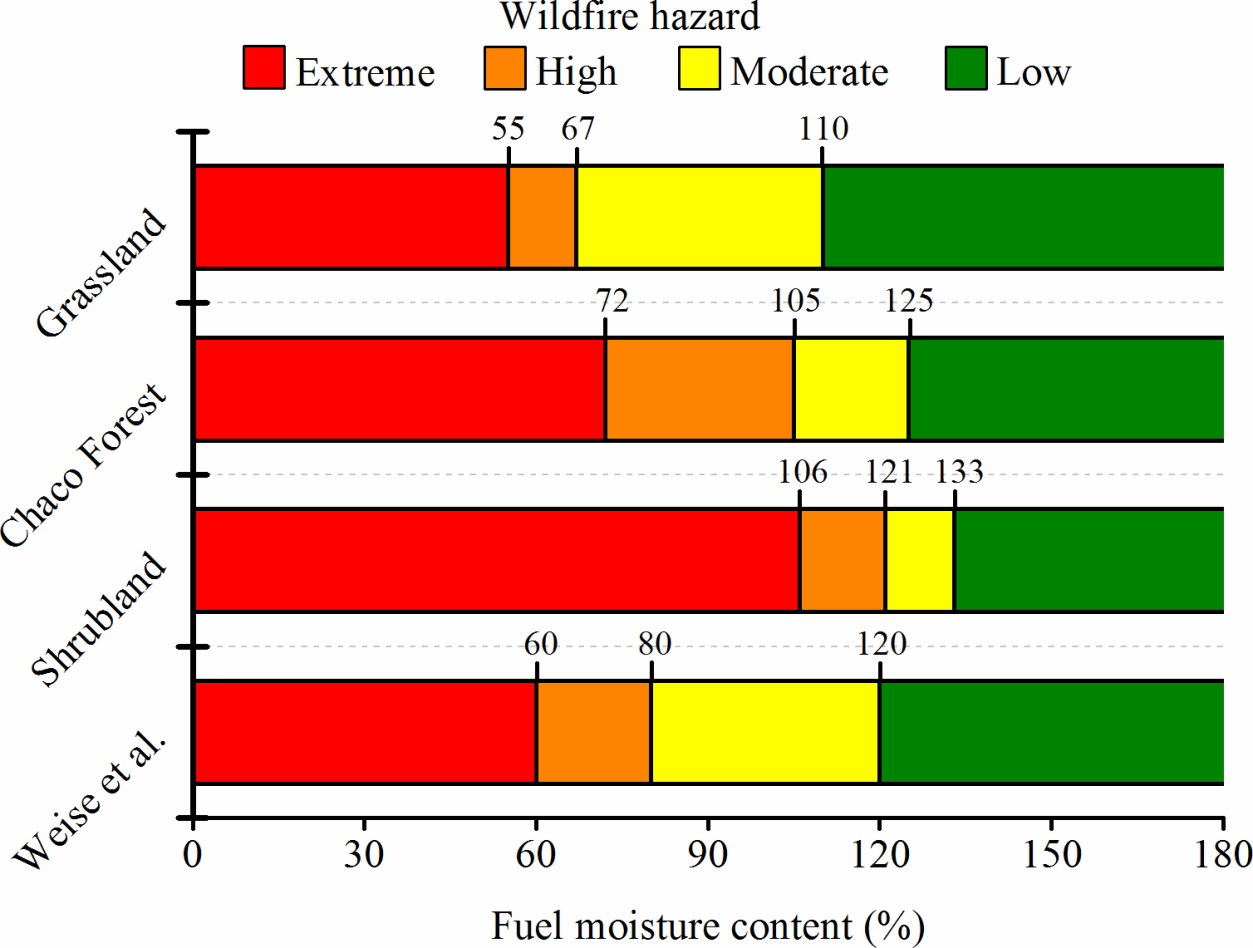
Fuel moisture thresholds determined to assess fire hazard in dominant land covers of the Sierras Chicas in Córdoba (Argentina) and its comparison with the thresholds established by Weise et al. [29]. In grasslands, FMC represents the whole fuelbed (mix of live and dead fuels) and in forests and shrublands FMC represents live fuels.

The fuel moisture thresholds that we established to assess wildfire hazard in our study area were considerably different among land covers (Fig 5). Grassland had the lowest FMC thresholds for all wildfire hazard categories, with fuelbed moisture content values of 55% and 67% defining the extreme and high wildfire hazard categories, respectively. In the case of forests, the thresholds for these categories were higher and are defined by LFMC values of 72% and 105%, respectively. Shrublands had the highest thresholds of all three land covers, with LFMC values of 106% and 121% defining the extreme and high wildfire hazard categories, respectively (Table 2; Fig 5).

When comparing our FMC thresholds with those proposed for live Chaparral by Weise et al. [29], we can see that our grassland thresholds are displaced downwards, which would be expected since we are including the whole fuelbed (mix of live and dead fuels). In the case of forests and shrublands, our thresholds are displaced upwards in comparison to those established by Weise et al. [29] (Fig 5). According to their hazard classification, as much as 75% of grasslands burned pixels, but only 36% and 22% for Chaco forests and shrublands, would have been included in the extreme fire hazard category, respectively (Table 3). When considering both high and extreme fire hazard categories, 92% of burned grasslands pixels and 69% of burned forest pixels would have been classified in these categories. Instead, only 32% of shrublands burned pixels would have been included in these categories and more than half burned pixels would have been classified as moderate fire hazard (Table 3).

**Table 3:** Comparison of the percentages of burned pixels in dominant land covers classified in the categories of fire hazard proposed by Weise et al. [29] based on live fuel moisture content and the percentages classified in each category according to our classification based on percentiles.

Fire hazard	Grasslands		Chaco forest		Shrublands		Our classification	
	%	% accum.	%	% accum.	%	% accum.	%	% accum.
**Extreme**	75	75	36	36	22	22	60	60
**High**	17	92	33	69	10	32	25	85
**Moderate**	6	98	25	94	53	85	12	97
**Low**	2	100	6	100	15	100	3	100

% accum.: is the accumulated percentage.

**Fig 6:**
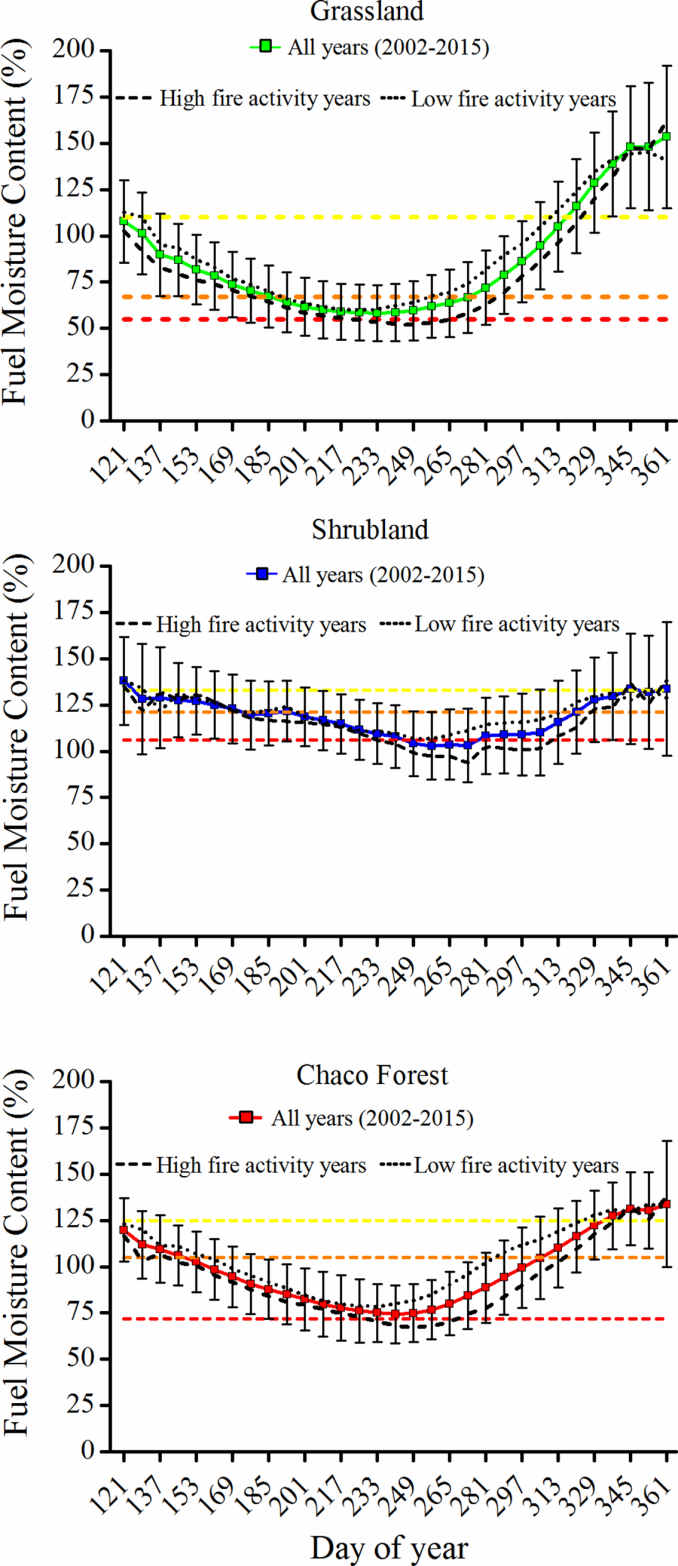
Average fuel moisture content (FMC) values through the fire season for different land cover types in the Sierras Chicas in Córdoba (Argentina) and their comparison with land cover specific fire hazard thresholds. Average FMC of all years included data from 2002 to 2015. Average FMC for high fire activity years (burned area higher than 3% of total area) included: 2003, 2006, 2008, 2009, 2011 and 2013, and average FMC for low fire activity (burned area lower than 1%) included: 2004, 2007, 2010, 2012, 2014 and 2015. The error bars represent average standard deviation. Dotted horizontal lines indicate the upper limit of extreme (red), high (orange) and moderate fire hazard thresholds (yellow). For reference, the first day of each month corresponds to the following days of year: 152 (June), 185 (July), 213 (August), 244 (September), 274 (October), 305 (November) and 335 (December).

The analysis of the evolution of average FMC for each land cover and date for all the years considered in our study showed that FMC decreased since the beginning of the fire season, reaching the minimum between August and September in all three land covers (Fig 6). The differences in average FMC of fire years vs. low fire years were less important during the first half of the fire season, but they increased considerably after September. The improvement of locally determining land cover specific FMC thresholds to assess wildfire hazard becomes evident when comparing average FMC values of all years vs. years with high fire activity. While the average FMC of all years was never below the extreme fire hazard threshold for grasslands and Chaco forests, during fire years FMC was below this threshold for 48 and 40 days, respectively. Also, in grasslands extreme and high fire hazard lasted 112 days in fire years vs. 80 days of the average. In shrublands, average FMC of all years was below the extreme fire hazard threshold for 32 days, but during fire years extreme hazard extended for 80 days (Fig 6). On average, FMC was 12.1, 11.4 and 7.1 percentage points lower in years with high fire activity than in years with low fire activity for Chaco forests, grasslands and shrublands, respectively.

## Discussion

In this study, we derived fuel moisture maps every 8 days at 500 m spatial resolution for fifteen fire seasons (2002–2016) in the Sierras Chicas in Córdoba (Chaco Serrano subregion of South America) using MODIS reflectance products. Then, we analyzed the relationship between pre-fire fuel moisture content and wildfire size; besides, we analyzed pre-fire FMC for different land covers in order to identify FMC thresholds to assess wildfire hazard. The differences in the pre-fire FMC distributions among land covers highlighted the necessity of determining land cover-specific FMC thresholds to assess wildfire hazard and local thresholds, instead of using those established for other ecoregions. To the best of our knowledge, this is the first study analyzing pre-fire FMC over several fire seasons in a non-Mediterranean ecosystem, aiming at assessing wildfire hazard.

Our results indicated that large fires occurred with lower fuel moisture content than small fires (median values: 74 vs. 91%), although the latter occurred over a wide range of FMC (Table 1). Similar to what we observed in the Sierras Chicas, large fires in the Chaparral in California take place when live fuel moisture lowers below a threshold of 79% [23], and small fires occur over a wide range of values [17]. When comparing the distributions of pre-fire fuel moisture content for shrublands and grasslands in our study area with those in the Mediterranean ecosystems (derived from satellite imagery using previously fitted empirical models [25]), we can see that fuels tend to burn with higher fuel moisture in the Sierras Chicas. While 50% of shrubland burned pixels had pre-fire FMC below 99%, the 50th pre-fire FMC percentile observed for Mediterranean shrubland was considerably lower (80%). In the case of grasslands, while the 50th percentile of pre-fire FMC was 52% in the Sierras Chicas, it corresponded to 26% fuel moisture for Mediterranean grasslands [25].

Our observation of small fires also occurring at low FMC is on the grounds that the occurrence of large fires depends on other variables as well. Weather conditions, such as wind speed, air temperature and relative humidity [23,34], fuel continuity [68], physiography [69] and suppression activities, all affect fire propagation and extent [70]. On the other hand, we found that higher FMC constrains the size reached by wildfires (Fig 3), which is in agreement with the general lack of fires after December (peak of the growing season). This lower flammability could be related to the higher proportion of water in plant tissues, which is associated with the beginning of the growing season triggered by seasonal rainfalls in our study area [15]. Nevertheless, these seasonal changes in fuel moisture could be linked to changes in foliar chemistry, also affecting fuel flammability, as observed in other studies [8,32,71]. Additionally, dead fuel loads and moisture might play a key role in determining fire behavior at the stand level [7,9,33,72], although dead fuel moisture in the Sierras Chicas does not vary considerably along the fire season [47].

Our FMC thresholds were sensitive to detect extreme fire hazard conditions during years with high fire activity in comparison to average conditions. Minimum FMC values were observed in August and September in all three land covers, which is when most large fires occur [36]. In the case of Chaco forests, our thresholds indicated high wildfire hazard for most of the fire season. However, considering that forests are less flammable than shrublands and grasslands [73], moisture alone seems not sufficient to represent true fire hazard in Chaco forests and it should be combined with other variables. Forest structure and fuel characteristics would be valuable metrics in this context due to their influence in forest flammability [74,75]. Shrublands reached extreme hazard conditions on average FMC circumstances (Fig 6). Considering that grass species are more flammable than shrub species [51] it is possible that our thresholds are slightly overestimating extreme hazard for shrublands. However, shrublands tend to have a high cover of herbaceous plants [15]; this means high dead fuel loads before the rainfall season, which might play an important role in governing initial flammability and preheat, dry and raise live fuels to combustion temperatures [11]. In addition, there are also other factors affecting flammability besides fuel moisture, such as heat content, chemical composition, arrangement of fuels in three dimensions, surface area-to volume ratio (SAV), fuel bed porosity, and fuel depth [51,76].

The fuelbed moisture thresholds we identified to assess wildfire hazard in grasslands were slightly lower than the thresholds proposed by Weise et al. [29]. Despite their thresholds were proposed for live fuels in the Chaparral, they seem quite adequate for assessing wildfire hazard for the grasslands in the Chaco Serrano subregion (Table 3). On the contrary, our hazard thresholds for forests and shrublands were considerably higher than Weise et al.'s [29], and based on their thresholds, as much as 68% of the burned shrublands belonged to the less hazardous categories, while this proportion reached 33% in the case of forests (Table 3). A better fire hazard assessment for forests and shrublands, as could be accomplished by applying our hazard thresholds to operational FMC maps in combination with other flammability metrics, is of special interest because together they account for 44% of the total burned area in our study area [36]. Moreover, forests provide highly valuable ecosystem services, and some shrubland physiognomies are the result of degrading processes affecting forests and have the potential to become forests again in the absence of disturbances. Wildfires might prevent the recovery of forest physiognomies especially when combined with livestock grazing [36,53].

Based on previous research on the dynamics of FMC in the Semiarid Chaco, the thresholds we identified in the Chaco Serrano grasslands might be useful to assess wildfire hazard in Semiarid Chaco grasslands, where fuelbed moisture content ranges between 10 and 240%, with an average of 46% [77]. On the contrary, the applicability of the thresholds we identified for the woody physiognomies in the Chaco Serrano subregion seems unlikely for the same physiognomies in the Semiarid Chaco, even acknowledging the limitations previously discussed. Maximum LFMC values observed in forests and shrublands in the Semiarid Chaco were below 50% and 60%, respectively [22], which is 10 percentage points below the minimum values measured for those physiognomies in the Chaco Serrano subregion [15]. This means that, according to our thresholds, Semiarid Chaco shrublands and forests would be classified as extreme fire hazard all year round.

Percentiles are often used to calibrate fire danger rating systems locally [65]. However, selecting the percentiles to define thresholds is somewhat subjective, but at the same time offers the advantage of selecting different percentiles according to the preferences and needs of different users. For instance, de Jong et al. [67] used the 99th percentile of historical FWI (Fire Weather Index of the Canadian Forest Fire Weather Rating System) data to determine exceptional fire weather conditions in the UK. In light of our results, which revealed differences in the shape of the distributions of pre-fire FMC for the different land covers (Fig 4), lower percentiles might be considered to establish fire hazard FMC thresholds for woody physiognomies.

It is also important to recall that FMC and derived wildfire hazard maps do not completely explain fire activity, since low FMC values are necessary, but not a cause of wildfires [13]. Integration of remote sensing derived FMC values with operational fire danger forecasting systems and other factors determining fire hazard will contribute to develop more complex and synergic fire hazard assessments [12,78,79]. In this context, factors related to ignition sources and conditions favoring fire spread, including weather conditions, should also be considered. Even with low FMC, the absence of ignitions will produce no fire. This means that the absence of fires can be predicted more reliably than fire activity, since above certain FMC thresholds, wildfires will not be sustained [13]. For instance, in the region, farmers illegally burn tussock grasses all year round (J.P.A, personal observation) but these fires do not develop into large wildfires after vegetation greens up in Spring.

In this study, we analyzed pre-fire FMC for different land covers in the Sierras Chicas in Córdoba, a landscape representative of the Chaco Serrano subregion of South America. The differences in the pre-fire FMC distributions among land covers highlighted the necessity of determining land cover-specific fuel moisture thresholds to assess wildfire hazard. We used percentiles to establish these thresholds and we chose our percentiles based on the consideration that a good wildfire hazard classification should include in its more hazardous categories most of the area burned. Our wildfire hazard classification applied to FMC maps derived from satellite imagery in an operational framework, together with other metrics of wildfire hazard, will contribute to improving early warning systems in our study area, that is home to more than 850,000 people and that is expected to grow considerably in the near future. At the moment, there is a project aiming at implementing MODIS derived FMC maps operationally, and the next step will be to derive wildfire hazard maps using our classification. These operational wildfire hazard maps will help to better allocation of fire protective resources to minimize negative impact on people, property and ecosystems.

## Supporting information

S1 Table Error matrix for the Land cover map of the Sierras Chicas (Córdoba, Argentina) derived from Landsat 8 OLI images (path/rows 229/81, 229/82) acquired on April 16 and August 6, 2013. (DOCX)

S2 Table Average pre-fire fuel moisture content (FMC) for fires of different sizes occurring from 2002 to 2016 in the Sierras Chicas in Córdoba, Argentina. (XLSX)

S3 Table Pre-fire fuel moisture content (FMC) for different land covers in the Sierras Chicas in Córdoba, Argentina. Data from 2002–2016.
(XLSX)

## References

[1] Bond WJ, Woodward FI, Midgley GF. The global distribution of ecosystems in a world without fire. New Phytol. 2005;165: 525–538. 10.1111/j.1469-8137.2004.01252.x 15720663

[2] Hantson S, Pueyo S, Chuvieco E. Global fire size distribution is driven by human impact and climate. Glob Ecol Biogeogr. 2015;24: 77–86. 10.1111/geb.12246

[3] Hawbaker TJ, Radeloff VC, Stewart SI, Hammer RB, Keuler NS, Clayton MK. Human and biophysical influences on fire occurrence in the United States. Ecol Appl. 2013;23: 565–582. 23734486 10.1890/12-1816.1

[4] Syphard AD, Radeloff VC, Hawbaker TJ, Stewart SI. Conservation Threats Due to Human-Caused Increases in Fire Frequency in Mediterranean-Climate Ecosystems. Conserv Biol. 2009;23: 758–769. 10.1111/j.1523-1739.2009.01223.x 22748094

[5] Bianchi LO, Defossé GE. Live fuel moisture content and leaf ignition of forest species in Andean Patagonia, Argentina. Int J Wildland Fire. 2015;24: 340–348. 10.1071/WF13099

[6] Rossa CG. The effect of fuel moisture content on the spread rate of forest fires in the absence of wind or slope. Int J Wildland Fire. 2017;26: 24–31.

[7] Weise DR, Zhou X, Sun L, Mahalingam S. Fire spread in chaparral—“go or no-go?” Int J Wildland Fire. 2005;14: 99–106.

[8] Melnik O. A proposed experimental methodology for assessing the effects of biophysical properties and energy content on live fuel flammability University of Alberta 2016.

[9] Cruz MG, Gould JS, Kidnie S, Bessell R, Nichols D, Slijepcevic A. Effects of curing on grassfires: II. Effect of grass senescence on the rate of fire spread. Int J Wildland Fire. 2015;24: 838 10.1071/WF14146

[10] Weise DR, Koo E, Zhou X, Mahalingam S, Morandini F, Balbi J-H. Fire spread in chaparral–a comparison of laboratory data and model predictions in burning live fuels. Int J Wildland Fire. 2016;25: 980 10.1071/WF15177

[11] Davies GM, Legg CJ, Smith AA, MacDonald AJ. Rate of spread of fires in Calluna vulgaris dominated moorlands. J Appl Ecol. 2009;46: 1054–1063. 10.1111/j.1365-2664.2009.01681.x

[12] Chowdhury EH, Hassan QK. Operational perspective of remote sensing-based forest fire danger forecasting systems. ISPRS J Photogramm Remote Sens. 2015;104: 224–236. 10.1016/j.isprsjprs.2014.03.011

[13] Chuvieco E, González I, Verdú F, Aguado I, Yebra M. Prediction of fire occurrence from live fuel moisture content measurements in a Mediterranean ecosystem. Int J Wildland Fire. 2009;18: 430 10.1071/WF08020

[14] Yebra M, Chuvico E, Riaño D. Estimation of live fuel moisture content from MODIS images for fire risk assessment. Agric For Meteorol. 2008;148: 523–536. 10.1016/j.agrformet.2007.12.005

[15] Argañaraz JP, Landi MA, Bravo SJ, Gavier-Pizarro GI, Scavuzzo CM, Bellis LM. Estimation of live fuel moisture content from MODIS images for fire danger assessment in Southern Gran Chaco. IEEE J Sel Top Appl Earth Obs Remote Sens. 2016;9: 5339–5349. 10.1109/JSTARS.2016.2575366

[16] Govender N, Trollope WS, Van Wilgen BW. The effect of fire season, fire frequency, rainfall and management on fire intensity in savanna vegetation in South Africa. J Appl Ecol. 2006;43: 748–758.

[17] Dennison PE, Moritz MA, Taylor RS. Evaluating predictive models of critical live fuel moisture in the Santa Monica Mountains, California. Int J Wildland Fire. 2008;17: 18–27. 10.1071/WF07017

[18] Qi Y, Dennison PE, Spencer J, Riano D. Monitoring live fuel moisture using soil moisture and remote sensing proxies. Fire Ecol. 2012;8: 71–87. 10.4996/fireecology.0803071

[19] Caccamo G, Chisholm LA, Bradstock RA, Puotinen ML, Pippen BG. Monitoring live fuel moisture content of heathland, shrubland and sclerophyll forest in south-eastern Australia using MODIS data. Int J Wildland Fire. 2012;21: 257–269. 10.1071/WF11024

[20] Pellizzaro G, Cesaraccio C, Duce P, Ventura A, Zara P. Relationships between seasonal patterns of live fuel moisture and meteorological drought indices for Mediterranean shrubland species. Int J Wildland Fire. 2007;16: 232–241. 10.1071/WF06081

[21] Viegas DX, Piñol J, Viegas MT, Ogaya R. Estimating live fine fuels moisture content using meteorologically-based indices. Int J Wildland Fire. 2001;10: 223–240.

[22] Kunst C, Ledesma R, Bravo S, Defossé G, Godoy J, Navarrete V. Dinámica de la humedad de los combustibles y su relación con la ecología y manejo de fuego, region chaqueña occidental (Argentina) II: follaje y residuos de árboles y arbustos. RIA Rev Investig Agropecu. 2014;40: 165–181.

[23] Dennison PE, Moritz MA. Critical live fuel moisture in chaparral ecosystems: a threshold for fire activity and its relationship to antecedent precipitation. Int J Wildland Fire. 2009;18: 1021–1027. 10.1071/WF08055

[24] Schoenberg FP, Peng R, Huang Z, Rundel PW. Detection of non-linearities in the dependence of burn area on fuel age and climatic variables. Int J Wildland Fire. 2003;12: 1–6.

[25] Jurdao S, Chuvieco E, Arevalillo JM. Modelling Fire Ignition Probability from Satellite Estimates of Live Fuel Moisture Content. Fire Ecol. 2012;7: 77–97. 10.4996/fireecology.0801077

[26] Barraza V, Grings F, Ferrazzoli P, Salvia M, Maas M, Rahmoune R, et al Monitoring vegetation moisture using passive microwave and optical indices in the Dry Chaco Forest, Argentina. IEEE J Sel Top Appl Earth Obs Remote Sens. 2014;7: 421–430. 10.1109/JSTARS.2013.2268011

[27] Jurdao S, Yebra M, Guerschman JP, Chuvieco E. Regional estimation of woodland moisture content by inverting Radiative Transfer Models. Remote Sens Environ. 2013;132: 59–70. 10.1016/j.rse.2013.01.004

[28] Peterson S, Roberts D, Dennison P. Mapping live fuel moisture with MODIS data: A multiple regression approach. Remote Sens Environ. 2008;112: 4272–4284. 10.1016/j.rse.2008.07.012

[29] Weise DR, Hartford RA, Mahaffey L. Assessing live fuel moisture for fire management applications. Fire in ecosystem management: shifting the paradigm from suppression to prescription Tall Timbers Fire Ecology Conference Proceedings. Tallahassee, FL.: Tall Timbers Research Station; 1998. Available: http://www.fs.fed.us/psw/publications/4403/psw_%3F%3F%3F%3F_weise000.pdf

[30] Scott JH, Thompson MP, Calkin DE. A wildfire risk assessment framework for land and resource management [Internet]. USDA Forest Service. Rocky Mountain Research Station; 2013 p. 83 Report No.: General Technical Report RMRS-GTR-315. Available: http://www.treesearch.fs.fed.us/pubs/44723

[31] Calkin DE, Cohen JD, Finney MA, Thompson MP. How risk management can prevent future wildfire disasters in the wildland-urban interface. Proc Natl Acad Sci. 2014;111: 746–751. 10.1073/pnas.1315088111 24344292 PMC3896199

[32] Jolly WM, Parsons RA, Hadlow AM, Cohn GM, McAllister SS, Popp JB, et al Relationships between moisture, chemistry, and ignition of Pinus contorta needles during the early stages of mountain pine beetle attack. For Ecol Manag. 2012;269: 52–59. 10.1016/j.foreco.2011.12.022

[33] Davies GM, Legg CJ. Fuel Moisture Thresholds in the Flammability of Calluna vulgaris. Fire Technol. 2011;47: 421–436. 10.1007/s10694-010-0162-0

[34] Dentoni MC, Defossé GE, Labraga JC, Del Valle HF. Atmospheric and fuel conditions related to the Puerto Madryn Fire of 21 January, 1994. Meteorol Appl. 2001;8: 361–370.

[35] Yebra M, Dennison PE, Chuvieco E, Riaño D, Zylstra P, Hunt ER, et al A global review of remote sensing of live fuel moisture content for fire danger assessment: Moving towards operational products. Remote Sens Environ. 2013;136: 455–468. 10.1016/j.rse.2013.05.029

[36] Argañaraz JP, Gavier Pizarro G, Zak M, Bellis LM. Fire regime, climate, and vegetation in the Sierras de Córdoba, Argentina. Fire Ecol. 2015;11: 55–73. 10.4996/fireecology.1101055

[37] Argañaraz JP, Radeloff VC, Bar-Massada A, Gavier-Pizarro GI, Scavuzzo CM, Bellis LM. Assessing wildfire exposure in the Wildland-Urban Interface area of the mountains of central Argentina. J Environ Manage. 2017;196: 499–510. 10.1016/j.jenvman.2017.03.058 28347968

[38] Albanesi S, Dardanelli S, Bellis LM. Effects of fire disturbance on bird communities and species of mountain Serrano forest in central Argentina. J For Res. 2014;19: 105–114. 10.1007/s10310-012-0388-4

[39] Gavier GI, Bucher EH. Deforestación de las Sierras Chicas de Córdoba (Argentina) en el período 1970–1997. Acad Nac Cienc Miscelánea. 2004;101: 1–27.

[40] Renison D, Cingolani AM, Suarez R. Efectos del fuego sobre un bosquecillo de Polylepis australis (Rosaceae) en las montañas de Córdoba, Argentina. Rev Chil Hist Nat. 2002;75: 719–727.

[41] Zak MR, Cabido M, Cáceres D, Díaz S. What Drives Accelerated Land Cover Change in Central Argentina? Synergistic Consequences of Climatic, Socioeconomic, and Technological Factors. Environ Manage. 2008;42: 181–189. 10.1007/s00267-008-9101-y 18427886

[42] Cingolani AM, Vaieretti MV, Giorgis MA, La Torre N, Whitworth-Hulse JI, Renison D. Can livestock and fires convert the sub-tropical mountain rangelands of central Argentina into a rocky desert? Rangel J. 2013;35: 285–297. 10.1071/RJ12095

[43] Bonansea M, Fernandez RL. Remote sensing of suspended solids concentration in a reservoir with frequent wildland fires on its watershed. Water Sci Technol. 2013;67: 217–223. 10.2166/wst.2012.560 23128642

[44] Van Wagner CE. Development and structure of the Canadian Forest Fire Weather Index System Ottawa: Canada Communication Group Publ; 1987.

[45] Noble IR, Gill AM, Bary GAV. McArthur’s fire-danger meters expressed as equations. Austral Ecol. 1980;5: 201–203.

[46] Bellis LM, Andreo V, Lighezzolo A, Argañaraz JP, Lanfri S, Clemoveki K, et al. Design and implementation of an operational early warning system of meteo fire risk based on geospatial data from remote sensing and numerical models. IGARSS 2015. Milán, Italia; 2015.

[47] Argañaraz JP. Dinámica espacial del fuego en las Sierras de Córdoba Universidad Nacional de Córdoba 2016.

[48] Argañaraz JP, Gavier Pizarro G, Zak M, Landi MA, Bellis LM. Human and biophysical drivers of fires in Semiarid Chaco mountains of Central Argentina. Sci Total Environ. 2015;520: 1–12. 10.1016/j.scitotenv.2015.02.081 25782079

[49] Cabido M, Zeballos SR, Zak M, Carranza ML, Giorgis MA, Cantero JJ, et al Native woody vegetation in central Argentina: Classification of Chaco and Espinal forests. Paruelo J, editor. Appl Veg Sci. 2018;21: 298–311. 10.1111/avsc.12369

[50] Giorgis MA, Cingolani AM, Chiarini F, Chiapella J, Barboza G, Ariza Espinar L, et al Composición florística del Bosque Chaqueño Serrano de la provincia de Córdoba, Argentina. Kurtziana. 2011;36: 9–43.

[51] Jaureguiberry P, Bertone G, Díaz S. Device for the standard measurement of shoot flammability in the field. Austral Ecol. 2011;36: 821–829. 10.1111/j.1442-9993.2010.02222.x

[52] Tálamo A, Caziani SM. Variation in woody vegetation among sites with different disturbance histories in the Argentine Chaco. For Ecol Manag. 2003;184: 79–92. 10.1016/S0378-1127(03)00150-6

[53] Giorgis MA, Cingolani AM, Cabido M. El efecto del fuego y las características topográficas sobre la vegetación y las propiedades del suelo en la zona de transición entre bosques y pastizales de las sierras de Córdoba, Argentina. Bol Soc Argent Botánica. 2013;48: 493–513.

[54] Gavier-Pizarro GI, Kuemmerle T, Hoyos LE, Stewart SI, Huebner CD, Keuler NS, et al Monitoring the invasion of an exotic tree (Ligustrum lucidum) from 1983 to 2006 with Landsat TM/ETM+ satellite data and Support Vector Machines in Córdoba, Argentina. Remote Sens Environ. 2012;122: 134–145. 10.1016/j.rse.2011.09.023

[55] Chladil MA, Nunez M. Assessing grassland moisture and biomass in Tasmania-the application of remote-sensing and empirical-models for a cloudy environment. Int J Wildland Fire. 1995;5: 165–171.

[56] Chuvieco E, Riaño D, Aguado I, Cocero D. Estimation of fuel moisture content from multitemporal analysis of Landsat Thematic Mapper reflectance data: Applications in fire danger assessment. Int J Remote Sens. 2002;23: 2145–2162. 10.1080/01431160110069818

[57] Davidson A, Wang S, Wilmshurst J. Remote sensing of grassland–shrubland vegetation water content in the shortwave domain. Int J Appl Earth Obs Geoinformation. 2006;8: 225–236. 10.1016/j.jag.2005.10.002

[58] Gander W, von Matt U. Smoothing Filters In: Gander W, Hřebíček J, editors. Solving problems in scientific computing using MAPLE and MATLAB. Springer Berlin Heidelberg; 1993 pp. 121–139.

[59] Jönsson P, Eklundh L. TIMESAT, a program for analyzing time-series of satellite sensor data. Comput Geosci. 2004;30: 833–845.

[60] Zak M. Patrones espaciales de la vegetación de la provincia de Córdoba Análisis complementario de información satelital y datos de campo. Universidad Nacional de Córdoba 2008.

[61] Meyer D, Dimitriadou E, Hornik K, Weignessel A, Leisch F, Chang C-C, et al. Misc functions of the Department of Statistics, Probability Theory Group (Formerly: E1071), TU Wien. 2015.

[62] R Core Team. R: a language and environment for statistical computing [Internet]. Vienna, Austria: R Foundation for Statistical Computing; 2017 Available: http://www.Rproject.org

[63] Hsu C-W, Chang C-C, Lin C-J. A practical guide to Support Vector Classification [Internet]. Taiwan: Department of Computer Science, National Taiwan University; 2010 Available: http://www.csie.ntu.edu.tw/~cjlin/papers/guide/guide.pdf

[64] Koenker R. quantreg: Quantile Regression R package version 5.05. Vienna: R Foundation; 2013.

[65] Andrews PL, Loftsgaarden DO, Bradshaw LS. Evaluation of fire danger rating indexes using logistic regression and percentile analysis. Int J Wildland Fire. 2003;12: 213–226.

[66] Heinsch FA, Andrews PL, Kurth LL. Implications of using percentiles to define fire danger levels. Proceedings of the 8th Symposium on Fire and Forest Meteorology. 2009.

[67] de Jong MC, Wooster MJ, Kitchen K, Manley C, Gazzard R, McCall FF. Calibration and evaluation of the Canadian Forest Fire Weather Index (FWI) System for improved wildland fire danger rating in the United Kingdom. Nat Hazards Earth Syst Sci. 2016;16: 1217–1237. 10.5194/nhess-16-1217-2016

[68] Turner MG, Hargrove WW, Gardner RH, Romme WH. Effects of fire on landscape heterogeneity in Yellowstone National Park, Wyoming. J Veg Sci. 1994;5: 731–742.

[69] McKenzie D, Miller C, Falk DA. Toward a theory of landscape fire In: McKenzie D, Miller C, Falk DA, editors. The Landscape Ecology of Fire. Springer Science & Business Media; 2011.

[70] de Torres Curth MI, Ghermandi L, Pfister G. Los incendios en el noroeste de la Patagonia: su relación con las condiciones meteorológicas y la presión antrópica a lo largo de 20 años. Ecol Austral. 2008;18: 153–167.

[71] McAllister S, Weise DR. Effects of Season on Ignition of Live Wildland Fuels Using the Forced Ignition and Flame Spread Test Apparatus. Combust Sci Technol. 2017;189: 231–247. 10.1080/00102202.2016.1206086

[72] Rossa CG, Fernandes PM. On the effect of live fuel moisture content on fire-spread rate. For Syst. 2017;26: eSC08 10.5424/fs/2017263-12019

[73] Landi MA. Caracterización del régimen de incendios, su relación con el clima y su efecto en la resiliencia y estructura de la vegetación Universidad Nacional de Córdoba 2018.

[74] Tiribelli F, Kitzberger T, Morales JM. Changes in vegetation structure and fuel characteristics along post-fire succession promote alternative stable states and positive fire-vegetation feedbacks. Collins B, editor. J Veg Sci. 2018;29: 147–156. 10.1111/jvs.12620

[75] Zylstra PJ. Flammability dynamics in the Australian Alps. Austral Ecol. 2018;43: 578–591. 10.1111/aec.12594

[76] Dibble AC, White RH, Lebow PK. Combustion characteristics of north-eastern USA vegetation tested in the cone calorimeter: invasive versus non-invasive plants. Int J Wildland Fire. 2007;16: 426–443. 10.1071/WF05103

[77] Kunst CR, Ledesma R, Bravo S, Defossé G, Godoy J, Navarrete V, et al Dinámica del contenido de humedad de pastos y su relación con la ecología del fuego en región chaqueña occidental (Argentina). Rev Investig Agropecu. 2015;41: 83–93.

[78] Chuvieco E, Aguado I, Jurdao S, Pettinari ML, Yebra M, Salas J, et al Integrating geospatial information into fire risk assessment. Int J Wildland Fire. 2014;23: 606–619. 10.1071/WF12052

[79] Argañaraz JP, Lighezzolo A, Clemoveki K, Bridera D, Scavuzzo JM, Bellis LM. Operational meteo fire danger system based on space information for Chaco Serrano. IEEE Lat Am Trans. 2018;16: 977–982.

