# Supplementary material for: Determining fuel moisture thresholds to assess wildfire hazard: A contribution to an operational early warning system
Source: PLoS One. 2018 Oct 4;13(10):e0204889. doi: 10.1371/journal.pone.0204889 (PMC6171885; doi:10.1371/journal.pone.0204889)
Supplement: S1 Table — (DOCX) [file pone.0204889.s001.docx]

**S1 Table. Error matrix for the Land cover map of the Sierras Chicas (Córdoba, Argentina) derived from Landsat 8 OLI images (path/rows 229/81, 229/82) acquired on April 16 and August 6, 2013.**

| Land cover classes |  | Reference data (pixels) | | | | | | | | |  | Accuracy | |
| --- | --- | --- | --- | --- | --- | --- | --- | --- | --- | --- | --- | --- | --- |
| Classification |  | GP | CF | Cr | Gr | R | Shr | Ur | Wt | Total |  | PA (%) | UA (%) |
| Glossy privet (GP) |  | 103 | 17 | 0 | 0 | 0 | 0 | 0 | 0 | 120 |  | 95.4 | 85.8 |
| Chaco Forest (CF) |  | 5 | 601 | 0 | 0 | 1 | 49 | 0 | 0 | 656 |  | 88.6 | 91.6 |
| Crops (Cr) |  | 0 | 0 | 746 | 3 | 3 | 0 | 0 | 0 | 752 |  | 95.9 | 99.2 |
| Grasslands (Gr) |  | 0 | 0 | 3 | 364 | 21 | 2 | 0 | 0 | 390 |  | 94.8 | 93.3 |
| Rocks (R) |  | 0 | 0 | 0 | 0 | 201 | 0 | 0 | 0 | 201 |  | 82.4 | 100 |
| Shrublands (Shr) |  | 0 | 60 | 5 | 17 | 2 | 590 | 0 | 0 | 674 |  | 92.0 | 87.5 |
| Urban (Ur) |  | 0 | 0 | 0 | 0 | 12 | 0 | 230 | 0 | 242 |  | 100 | 95.0 |
| Water (Wt) |  | 0 | 0 | 0 | 0 | 4 | 0 | 0 | 281 | 285 |  | 100 | 98.6 |
| Total |  | 108 | 678 | 754 | 384 | 244 | 641 | 23 | 281 | 3320 |  |  |  |

Global precision: 93.8 % (3116/3320 pixels). PA: Producer’s accuracy. UA: User’s accuracy.

According to our land cover map, shrublands cover an area of 339,500 ha (41.8 % of the study area), followed by crops: 219,400 ha (27.0 %), grasslands: 116,000 ha (14.3 %), Chaco forest: 94,900 ha (11.7 %), urban areas: 19,160 ha (2.4 %), water bodies: 9,000 ha (1.1 %), Rock: 8,450 ha (1.0 %) and Glossy Privet: 3,600 ha (0.4 %).
